# Isolated vocal tremor as a focal phenotype of essential tremor: a retrospective case review

**DOI:** 10.1186/s40734-015-0016-5

**Published:** 2015-03-02

**Authors:** Amar Patel, Steven J Frucht

**Affiliations:** Department of Neurology, Icahn School of Medicine at Mount Sinai, 5 E 98th Street, New York, NY 10029 USA

**Keywords:** Vocal tremor, Isolated vocal tremor, Essential tremor, Pharyngeal tremor, Sodium oxybate

## Abstract

**Background:**

Essential tremor (ET) is a common condition associated with significant physical and psychosocial disability. “Classic” ET is a clinical syndrome of action tremor in the upper limbs and less commonly the head, jaw, voice, trunk, or lower limbs. Current diagnostic criteria for ET exclude isolated vocal tremor (IVT). Failure to recognize IVT as a form of ET may contribute to misdiagnosis and missed opportunities for treatment.

**Methods:**

We conducted a retrospective review of cases referred for voice disturbance. Patients with a primary diagnosis of vocal tremor were included while those with a diagnosis of spasmodic dysphonia where excluded.

**Results:**

19 cases of vocal tremor were identified, of which 17 patients (89%) were female. The average age of vocal symptom onset was 64 (SD 8.0) and patients had been symptomatic an average of 6 years (SD 4) at their initial visit. 8 patients had IVT while 11 also had evidence of subtle head or limb tremor. 8 patients (42%) had a family history of ET, with vocal tremor specifically identified in 5 of those cases (26%). 11 patients (58%) noted transient tremor improvement after alcohol consumption. Primidone and propranolol were the most common medications prescribed to these patients prior to consultation. 7 patients were given a trial of 1 gm of sodium oxybate in the office as part of a clinical trial, with at least mild improvement in vocal tremor noted by qualitative assessment.

**Conclusions:**

ET may present as vocal tremor with little or no associated limb tremor. It may be a more common manifestation of ET in women. A family history of tremor and improvement in tremor after consuming alcohol can often be elicited on history. We propose that IVT may be part of the spectrum of ET.

**Electronic supplementary material:**

The online version of this article (doi:10.1186/s40734-015-0016-5) contains supplementary material, which is available to authorized users.

## Background

Essential tremor (ET) is the most common pathological cause of tremor, defined as a rhythmic, oscillatory movement of a body part about an axis. ET is the most common movement disorder in the general population and of those presenting for neurological evaluation [1]. Many cases of ET are familial, with anywhere from 17% to 100% cited in various studies, and inherited most likely in an autosomal dominant fashion with variable penetrance [2,3]. The tremor of ET occurs during voluntary movement, posture and action [4]. ET has been defined by the Movement Disorder Society Consensus Statement on Tremor as a “bilateral, largely symmetric postural or kinetic tremor involving hands and forearms that is visible and persistent” [5]. These criteria were based on Tremor Investigation Group guidelines which also included duration of illness as a major criterion [6]. Other causes of tremor, e.g. dystonia, must be excluded, and although traditionally vocal tremor is recognized as a feature of ET in anywhere from 10 to 25% of cases, isolated vocal tremor (IVT), defined as tremor of the voice in the absence of other observable tremor, does not meet current criteria for ET [7]. Voice tremor has thus classically been considered a secondary feature of ET which does not occur without upper limb tremor.

Here we describe a series of cases in which tremor of the phonatory apparatus is the sole or predominant manifestation of ET. We also compare the current series to previously described essential voice tremor populations. We propose that the phenomenologic characteristics of isolated vocal tremor (IVT) are consistent with its inclusion as a focal form of ET; and where both vocal and limb tremor occur, the former need not be a secondary feature of the latter, but rather the primary concern of the patient.

## Methods

We conducted a retrospective review of the medical records of patients referred to the senior author for voice disturbance over a ten-year period. Patients with a diagnosis of vocal tremor were included, while those with a diagnosis of spasmodic dysphonia where excluded. The study was approved by the Institutional Review Board of Mount Sinai School of Medicine. Written informed consent was obtained for the use of video examinations. A waiver of consent for review of medical records was granted by the IRB.

## Results

19 patients with a primary diagnosis of vocal tremor were evaluated between 2003 and 2013 (Table 1). 17 patients (89%) were female. The average age of onset of vocal difficulty was 64 (SD 8.0). Patients had been symptomatic an average of 6 years (SD 4) upon their initial consultation. 8 patients (42%) had a family history of ET, with vocal tremor specifically identified in 5 of those cases (26%). 11 patients (58%) noted transient tremor improvement after alcohol consumption; the remaining patients did not drink alcohol. On examination, 17 patients (89%) had a visible tremor of the pharyngeal musculature when speaking. Sustained vowel (A and E) phonation most reliably brought out the rhythmic oscillations of voice intensity at a frequency of approximately 4–8 Hz. Supplementary patient videos show this in greater detail for a sample of the patients described in this case series [see Additional file 1: Videos S1 and S2 and Additional file 2: Video S3]. All 9 patients who were referred for or had prior videostroboscopic evaluation of the vocal cords had visual confirmation of vocal tremor. 6 patients (32%) were noted to have very slight head tremor while 3 patients (16%) had very mild action tremor of the upper extremities. Two patients had both mild head and limb tremor. Of these 11 patients with vocal tremor plus tremor elsewhere, 4 had videostroboscopic evaluation of the vocal cords that was more consistent with vocal tremor rather than spasmodic dysphonia. Rest tremor of the upper limbs was present in only one patient, and none had signs of rigidity or bradykinesia to support a diagnosis of parkinsonism. No patient had sought medical treatment of their head or limb tremor prior to consultation for their voice tremor. Primidone and propranolol were the most common medications prescribed to these patients prior to consultation. 7 patients were treated with 1 gm of the alcohol analogue sodium oxybate after their consultation as part of a separate IRB-approved clinical trial. All 7 patients were observed to have at least mild improvement in vocal tremor, as noted by a reduction in the amplitude of the tremor without a change in its frequency. Patients were subsequently followed for as little as 6 months and as long as 10 years without the development of dystonia or parkinsonism.

**Table 1:** **Case series**

**Case**	**Gender**	**Age of onset**	**Duration (years)**	**Family history**	**Alcohol response**	**Medications**	**Head tremor**	**Kinetic arm tremor**	**Rest tremor**	**Video-stroboscopy**	**Visible palatal or pharyngeal muscle tremor**	**Response to 1 gm sodium oxybate**
1	F	56	4	No	Unaware	Primidone (mild response, side effects)	No	No	No	Yes	Yes	N/A
2	F	71	3	No	Yes	None	Slight	Slight	No	Yes	Not commented upon	N/A
3	F	64	2	Vocal tremor (Mother)	Unaware	None	Slight	No	No	No	Yes	N/A
4	F	70	10	No	Unaware	Lorazepam (slight improvement)	Slight	No	No	No	Yes	N/A
5	F	69	2	No	Unaware	Atenolol (No response)	Slight	No	No	Yes	Yes	N/A
6	F	87	1	No	Unaware	Lorazepam (No Response)	Slight	No	No	No	Yes	N/A
7	F	54	15	Parkinson	Yes	None	No	Slight	No	No	Yes	Mild improvement
8	F	69	3	Action tremor (Mother), Vocal tremor (Sister)	Yes	Mirapex (No response); Primidone (No Response)	No	Slight	No	Yes	Not Commented Upon	Mild improvement
9	F	56	5	Vocal tremor (Mother, Sister)	Yes	Propranolol (No Response)	No	No	No	Yes	Yes	Mild improvement
10	F	64	6	No	Unaware	Propranolol (No Response)	No	No	No	Yes	Yes	Mild improvement
11	M	72	15	No	Yes	None	Slight	No	No	No	Yes	Mild improvement
12	F	63	2	Action tremor (Father)	Yes	Primidone (Unknown Response)	No	No	No	No	Yes	N/A
13	F	66	3	Vocal tremor (Mother)	Unaware	Primidone (Mild improvement, Yes side effects)	No	Slight	No	No	Yes	N/A
14	F	45	20	Action tremor (Father, Son)	Yes	Primidone (Moderate improvement)	Slight	No	Slight	No	Yes	N/A
15	F	70	2	No	Unaware	None	No	No	No	No	Yes	N/A
16	F	81	3	No	Yes	Primidone (Moderate improvement)	No	No	No	No	Yes	N/A
17	M	56	6	Tremor (Father, Paternal Aunt/Uncle/Grandmother	Yes	Propranolol (Mild improvement)	No	No	No	Yes	Yes	Mild improvement
18	F	65	9	No	Yes	Propranolol, Primidone (Mild improvement)	Slight	Slight	No	Yes	Yes	Mild improvement
19	F	45	2	Action tremor (Father), Vocal tremor (Paternal Aunt)	Yes	Propranolol (Moderate improvement)	No	No	No	Yes	Yes	N/A

## Discussion

The present case series provides some comparisons and contrasts with previously reported populations of voice tremor (Table 2). In agreement with previous reports is the finding that voice tremor is a condition of the elderly. The largest study population of voice tremor patients included only two cases of voice tremor presenting in the second and third decade of life [8]. This matches the association of another “midline” symptom, head tremor, with older age independent of duration of tremor [9]. Although epidemiological studies of ET find an equal gender distribution [10], voice tremor seems to affect more women, who represent approximately 80% of the patients identified in the literature. This corresponds with a similar gender bias in head tremor [9], suggesting that older age and female gender are defining characteristics of midline ET symptoms. Given the relatively small number of patients in some of these case series selection bias is possible, and a population-based study of essential voice tremor would be needed to more definitively address the question. Rates of familial voice tremor appear to be similar to those of familial limb tremor, although the heterogeneity of inheritance rates and patterns in the latter make this comparison less useful clinically [2].

**Table 2:** **Vocal tremor case series comparative analysis**

**Study**	**Year**	**No. of cases**	**Mean age**	**Mean duration of symptoms (years)**	**Female (%)**	**Family history (%)**	**Isolated vocal tremor (%)**	**Alcohol response (%)**
Brown et al. [11]	1963	31			55	52	19	
Koller et al. [12]	1985	7	64	16	14	57	14	
Busenbark et al. [13]	1996	9	73	32	89			
Hertegard et al. [14]	2000	15	73		87			
Warrick et al. [15]	2000	10	64	12	80	20		40
Adler et al. [16]	2004	13	73		85	38	54	
Bove et al. [17]	2006	20	66	8	55	45		45
Sulica and Louis [8]	2010	34	70	7	93	38	56	27
Patel and Frucht	2014	19	64	6	89	42	42	58

A significant proportion of patients in the present case series reported transient tremor improvement after drinking alcohol, at rates similar to other studies of voice tremor. In fact, all alcohol “non-responders” were unaware of their response due to reported abstention from alcohol. This would suggest that an even greater percentage of voice tremor cases would respond if challenged with alcohol and objectively measured. Such a high percentage of voice tremor response to alcohol is in line with reported rates of response of limb tremor in those patients aware of the effect of alcohol on tremor [18]. Given the robust response to alcohol, it is not surprising that all patients treated with sodium oxybate had at least a mild reported improvement in voice tremor. Sodium oxybate is the salt form of γ-hydroxybutyric acid. Mice deficient in GABA_a_ receptor have an ET-like tremor that improves with ethanol, suggesting a GABAergic mechanism in ET [19]. A previous open-label, single-blinded trial of sodium oxybate with medically refractory ET showed a dose-dependent improvement in tremor ratings [20]. Three of the patients in the current case series who responded to sodium oxybate had previously had no response to primidone and propranolol. Proper diagnosis of voice tremor can thus lead to alternative medication strategies for this often refractory condition.

Our case series supports the findings of more recent studies of voice tremor which suggest that voice tremor can occur in isolation or with limb tremor of little or no consequence. Sulica and Louis determined that 56% of patients in their voice tremor study had arm tremor within the range observed in similarly matched aged controls, as determined by the WHIGET scale [8]. Our retrospective review found a similar percentage of patients without clinically significant limb tremor through a qualitative assessment. In our series, the 8 patients with IVT and 11 patients with vocal tremor plus head/limb tremor showed similar characteristics of age, gender, and alcohol responsivity. The historical categorization of voice tremor as a secondary feature of ET thus likely contributes to the under-recognition of essential voice tremor in clinical practice. Further longitudinal studies are needed to determine if these IVT patients develop tremor elsewhere or better resemble classic ET patients over time.

At the same time, it is important to recognize that inclusion of IVT in groups of ET patients may not be helpful when investigating the etiology and pathogenesis of ET. Indeed, emerging understanding of the broad heterogeneity of ET, which IVT adds to, suggests that ET may be a family of disorders rather than a single entity. This has significant research implications for correctly identifying disease risk factors, prognostic markers, and future clinical trial design which may be specific to the “type” of ET studied [21].

Limitations of our retrospective review include the lack of electrophysiologic characterization of voice and limb tremor. Similarly, not all patients had videostroboscopic evaluation. Mild cases of adductor spasmodic dysphonia may be misdiagnosed as essential voice tremor. Without electromyographic evaluation of the vocal cords or limbs, subtle dystonic tremor which appears regular can mimic essential tremor on qualitative evaluation [22]. However, the similar age of onset, rates of familial tremor, lack of other dystonic features (phoneme-specific voice breaks, sensory geste antagoniste), and robust alcohol response in our cases compared with known ET characteristics all suggest that misdiagnosis in these cases was less likely. Further confounding this distinction between dystonia and ET is the possibility that involuntary movements like dystonia may co-occur in long-standing “pure” ET patients; raising the question as to whether such patients have two diseases or a secondary feature of a singular condition [23]. Indeed, patient 1 in Additional file 1: Video S1 has a slight head tilt during singing and a co-occuring cervical dystonia may not be excluded by exam alone.

## Conclusions

This retrospective review adds to the growing understanding of essential voice tremor as part of the heterogeneous presentation of ET. Recognition of IVT occurring in the absence of “classic” ET features may aid in improving diagnosis and identifying new therapeutics for this functionally disabling and often medically refractory condition.

## Additional files

Additional file 1: Videos S1 and S2. Isolated vocal tremor 1, Isolated vocal tremor 2, Isolated vocal tremor 3. Legend: Sustained vowel (A and E) phonation most reliably brings out the vocal tremor. Singing or changing pitch does not improve the tremor, unlike in cases of spasmodic dysphonia. The patients had a similar frequency tremor during normal spontaneous speech (not shown). Videostroboscopic examination of both patients performed by ENT consultant was consistent with vocal cord tremor rather than spasmodic dysphonia. Additional file 2: Video S3. Legend: Patient 1 manifests a tremor of the pharyngeal musculature which can be easily observed by the examiner. Patient 2 displays a prominent tremor of the soft palate. Laryngoscopy and videostroboscopic evaluations of patient 2 were more consistent with vocal tremor than spasmodic dysphonia.

## Abbreviations

IVT: Isolated vocal tremor
ET: Essential tremor
